# Flecainide Toxicity From Clinical Pharmacology Perspectives

**DOI:** 10.7759/cureus.65884

**Published:** 2024-07-31

**Authors:** Naoyuki Otani, Hirokazu Wakuda, Ichiro Oikawa, Naoto Uemura, Takanori Yasu

**Affiliations:** 1 Cardiology, Dokkyo Medical University Nikko Medical Center, Nikko, JPN; 2 Clinical Pharmacology and Therapeutics, Faculty of Medicine, Oita University, Yufu, JPN; 3 Cardiology, Yonago Medical Center, Yonago, JPN; 4 Cardiovascular Medicine and Nephrology, Dokkyo Medical University Nikko Medical Center, Nikko, JPN

**Keywords:** therapeutic drug monitoring, ckd (chronic kidney disease), toxicity, flecainide, arrhythmia

## Abstract

We report a case comparing the measured half-life of flecainide with the half-life stated on the label. An 84-year-old woman presented with symptoms of anorexia and exertional dyspnea. She had undergone mitral and aortic valve replacements and excision of the membranous septum in the atrium for mitral and aortic stenosis and cor triatriatum. She was regularly administered 100 mg/day flecainide for paroxysmal atrial fibrillation. A previous electrocardiogram (ECG) showed a regular sinus rhythm. However, upon admission, the ECG revealed a heart rate of 94 bpm and an accelerated idioventricular rhythm originating from the left ventricle. Flecainide toxicity was suspected, leading to the discontinuation of flecainide treatment. The following day, the serum flecainide concentration was 1,348 ng/mL, exceeding the therapeutic window of 200-1,000 ng/mL. After discontinuing flecainide, the accelerated idioventricular rhythm ceased, and a regular sinus rhythm temporarily returned. We measured blood drug concentrations several times; our calculated half-life was 56.8 h, approximately five times longer than the half-life of 11.0 h stated on the package insert. To ensure safe and effective therapy with antiarrhythmic drugs, prioritizing therapeutic drug monitoring and carefully monitoring pharmacokinetics is important, particularly during the elimination phase.

## Introduction

Each patient's medical condition is unique, and effective drug therapy necessitates an understanding of how an individual's condition influences pharmacokinetics. Pharmacokinetic parameters, such as volume of distribution and clearance, can vary significantly between patients, even when the same dose is administered. The volume of distribution, a theoretical value, is the volume of bodily fluids required to dilute a drug to the same concentration in the blood that would be obtained if the drug were received in the body. The volume of distribution multiplied by the concentration of the drug in the body is calculated as the amount of the drug in the body after administration. In other words, a large volume of distribution indicates that the drug disperses widely in body tissues, making eliminating it challenging. Conversely, clearance reflects how quickly the body removes a drug per unit of blood concentration. Regarding the same blood concentration, the higher the clearance value, the faster the drug is eliminated from the body. Total clearance encompasses both renal clearance, which signifies the elimination of drugs through the kidneys, and extrarenal clearance, which denotes elimination from organs besides the kidneys, such as the liver. Clearance varies widely with age, body size, and renal/hepatic function, resulting in individual differences in blood drug concentrations [1].

Although antiarrhythmic drugs have powerful pharmacological effects, they also have serious adverse effects and a narrow safety margin. To ensure safe and effective drug therapy with such medications, the development of quantitative methods to accurately evaluate and predict their pharmacokinetics is necessary. Considering drugs whose therapeutic effects are closely related to the serum concentrations, therapeutic drug monitoring (TDM) is adopted, which involves measuring the serum blood concentrations of the drug and designing dosages using pharmacokinetic analyses. We encountered a case where a patient on a fixed, low dose of flecainide experienced adverse events as the renal function declined, resulting in blood levels that were above the therapeutic window. Although rigorous TDM was not performed in this patient, we estimated the patient’s characteristic pharmacokinetics based on measurable post-discontinuation serum concentrations, thereby confirming the importance of TDM in such patients.

## Case presentation

An 84-year-old woman presented to the emergency department at night with anorexia and shortness of breath on exertion. Nine years prior, she had undergone mitral and aortic valve replacements and excision of the membranous septum in the atrium for mitral and aortic stenosis and cor triatriatum. Postoperatively, flecainide treatment was initiated for paroxysmal atrial fibrillation. The patient presented with stage 3b chronic kidney disease (CKD); flecainide was adjusted from the standard dose of 200 mg/day to 100 mg/day, and sinus rhythm was maintained. At the time of that outpatient visit, approximately one year before admission, the serum creatinine concentration was 1.06 mg/dL, and the estimated glomerular filtration rate was 37.8 mL/min/1.73 m^2^.

**Figure 1:**
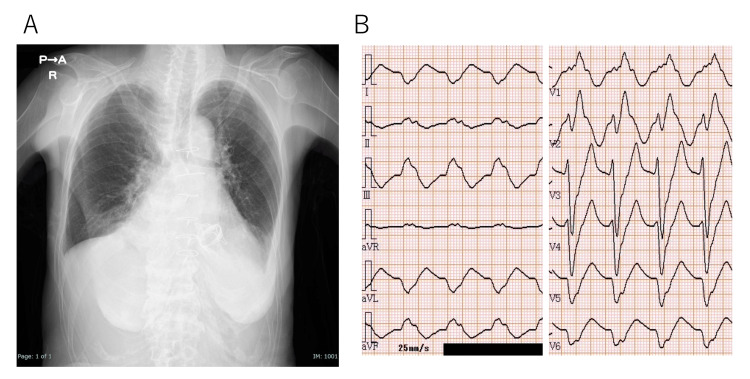
Chest radiography and electrocardiogram on admission On admission, chest radiography reveals an increased cardiothoracic ratio (A). A 12-lead electrocardiogram of an 84-year-old woman on admission shows a heart rate of 94 bpm, right bundle branch block type, and rS pattern in V6, without preceding P waves, revealing an accelerated idioventricular rhythm of left ventricular origin (B).

She reported a 10-day history of loss of appetite and shortness of breath on exertion. Her level of consciousness was normal. She was a petite woman with a height of 152.0 cm, weight of 28.9 kg, and body mass index of 12.5 kg/m^2^. Her vital signs were as follows: body temperature: 36.3℃; blood pressure: 98/70 mmHg; pulse rate: 95 beats/min; and peripheral capillary oxygen saturation: 95% on room air. Physical examination revealed a pansystolic murmur with the apex as the strongest point, graded as Levine V/VI, and a third heart sound. No rales were heard in either lung. Jugular vein distension was observed in the supine position and the liver was palpable. No leg edema was noted. Blood biochemical findings included a serum creatinine concentration of 1.85 mg/dL, an estimated glomerular filtration rate of 20.5 mL/min/1.73 m^2^, and a CKD severity classification of stage 4 (Table 1). On admission, chest radiography revealed an enlarged heart (Figure. 1A), and echocardiography showed a left ventricular ejection fraction of 75%, a cardiac output index of 1.6 L/min/m^2^, mitral regurgitation (moderate-to-severe), tricuspid regurgitation (moderate-to-severe), and a tricuspid regurgitation pressure gradient of 46 mmHg. The electrocardiogram (ECG) showed a heart rate of 94 bpm, right bundle branch block type, and rS pattern in V6 without preceding P waves, indicating an accelerated idioventricular rhythm of left ventricular origin, essentially a slow ventricular tachycardia (Figure 1B). We suspected that this ECG pattern was due to the enhanced sodium channel-blocking effect of flecainide, leading to a conduction delay in the ventricle. Therefore, flecainide toxicity was suspected.

**Table 1:** Laboratory tests on admission

Lab parameters	Observed value	Reference range
Biochemistry		
Total protein	7.1 g/dl	6.6-8.1 g/dl
Albumin	3.3 g/dl	4.1-5.1 g/dl
Aspartate aminotransferase	137 U/L	13-30 U/L
Alanine aminotransferase	32 U/L	7-23 U/L
Gamma glutamyl transpeptidase	85 U/L	9-32 U/L
Total bilirubin	1.6 mg/dl	0.4-1.5 mg/dl
Blood urea nitrogen	55.2 mg/dl	8-20 mg/dl
Sodium	137 mEq/L	138-145 mEq/L
Potassium	6.1 mEq/L	3.6-4.8 mEq/L
Chloride	95 mEq/L	101-108 mEq/L
Creatinine	1.85 mg/dl	0.46-0.79 mg/dl
Estimated glomerular filtration rate (eGFR)	20.5 ml/min/1.73 m^2^	
Serology		
Brain natriuretic peptide	1582.4 pg/ml	<18.4 pg/ml
Blood count		
White blood cells	7510 /μl	3500-9100 /μl
Hemoglobin	11.3 g/dl	11.3-15.2 g/dl
Hematocrit	35%	33.4-44.9%
Platelets	14.9×10^4^ /μl	13.0-36.9×10^4^ /μl

**Figure 2:**
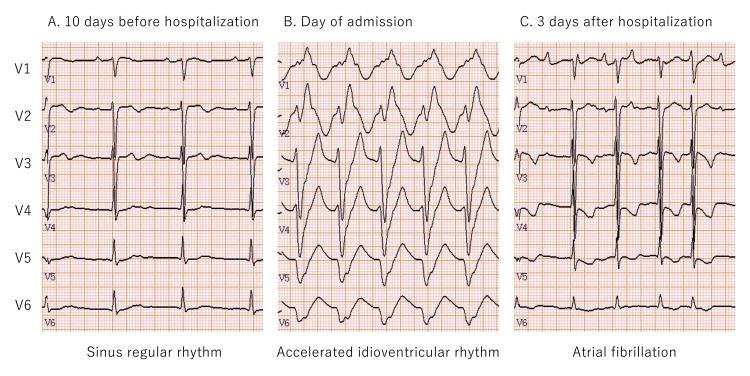
Time course of electrocardiogram Comparison of ECG in six chest leads (V1-V6). The ECG 10 days before admission shows sinus regular rhythm (A). The ECG on the day of admission shows accelerated idioventricular rhythm (B). The ECG three days after admission shows atrial fibrillation (C). ECG: electrocardiogram

She took flecainide between 6:00 and 8:00 AM on the day of her visit but stopped taking it in the evening after a visit to the emergency room of our hospital at around 7:30 PM. The next morning, the serum flecainide concentration was measured at around 6:30 AM. Approximately 24 h after her last dose, her blood flecainide concentration was 1,348 ng/mL. The therapeutic plasma concentrations of flecainide typically range between approximately 200 and 1,000 ng/mL. Figure 2 shows the time course of the 6-lead ECG comparisons in the chest. Three days after admission, the accelerated idioventricular rhythm resolved. The patient’s ECG temporarily exhibited sinus rhythm. However, it later transitioned into atrial fibrillation (Figure 2C). As the serum flecainide concentration had decreased, she was discharged from the hospital.

**Figure 3:**
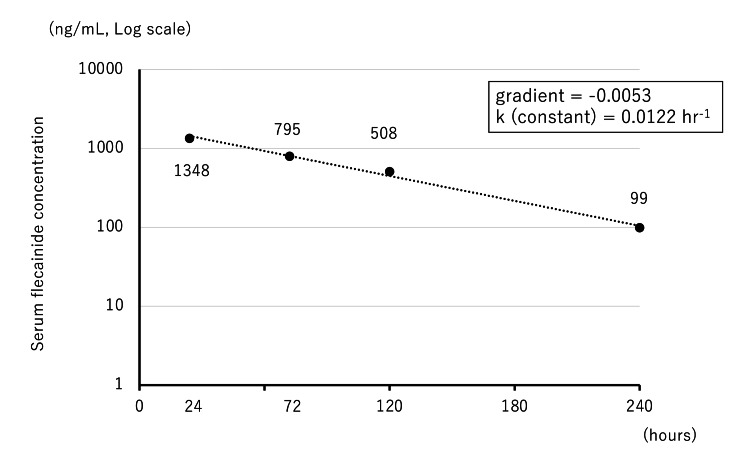
Time course of serum flecainide concentrations Time course of serum flecainide concentrations since admission to the hospital. The serum flecainide concentration is converted to a logarithm, and the gradient and constant are calculated. Based on the serum flecainide concentrations, the half-life is calculated to be 56.8 h.

Serum flecainide concentrations were measured and calculated at 24, 72, 120, 240, and 336 h after the last dose (0 h). The concentration at 336 h was below the sensitivity (49 ng/mL or less). The elimination rate constant (k), which represents the slope of the relationship of the time course with serum flecainide concentration, was estimated to be approximately 0.0122 hr^−1^, yielding a calculated half-life of 56.8 h, 0.693 divided by k (Figure 3). According to the Japanese package insert, the half-life of flecainide is 11 ± 3 h. Our measured half-life was approximately five-fold longer than that listed on the package insert.

## Discussion

Flecainide is an antiarrhythmic drug with sodium channel-blocking activity and is currently used mainly for supraventricular arrhythmias such as atrial fibrillation. Since elevated serum flecainide concentrations increase the risk of developing cardiovascular events, maintaining serum flecainide concentrations within the therapeutic window is important. However, serum flecainide concentrations vary widely among individuals; some patients may experience increased levels even with appropriate dosage and administration. Therefore, establishing an appropriate dosage through TDM is essential. In patients with severe renal impairment, characterized by a creatinine clearance rate of less than 20 mL/min, the drug dosage is reduced to 100 mg/day. This adjustment is necessary as blood concentrations in these individuals may exceed predicted levels. The effective blood concentration is 200-1000 ng/mL, yet the serum flecainide concentration in our patient reached up to 1348 ng/mL. Utilizing the values acquired from the TDM samples, we calculated the half-life of flecainide. Comparing it with the half-life specified in the package insert, we observed a disparity of approximately five-fold. The following factors may be responsible for this:

1) The severity of our patient’s CKD progressed from stage 3b to stage 4. Challenges in managing her heart failure posed a significant hurdle, further complicated by her recent evaluation via cardiac catheterization to assess cardiac function. During this procedure, a small amount of contrast medium was administered, which, we suspect, may have compromised her already impaired renal function. In addition, the patient was experiencing anorexia, which might have led to reduced fluid levels. This decreased fluid intake could have contributed to a reduction in extracellular fluid volume and subsequently diminished renal blood flow. We hypothesized that the rapid change in kidney function resulted in decreased renal clearance of flecainide, consequently elevating blood drug concentrations. Conard et al. reported that following a single 200 mg oral dose, plasma half-life averages 17 h in individuals with moderate renal failure and 26 h in those with end-stage disease. Correspondingly, plasma clearance averages 6.7 and 5.1 mL/min/kg in the respective groups. These data indicate that flecainide elimination from plasma is slower in patients with renal disease [2,3].

2) Conard et al. reported that the rate of flecainide elimination from the plasma in patients with congestive heart failure is slower compared with that in healthy volunteers. They reported that plasma half-life averages 19 h in patients with congestive heart failure and 14 h for healthy volunteers; correspondingly, plasma clearance averages 8.1 and 10.2 mL/min/kg in patients and healthy volunteers [2]. Although decreased cardiac output due to cardiac failure may reduce renal blood flow and potentially affect renal clearance, the nearly identical cardiac output index observed in the previous and current echocardiograms in our patient suggests that cardiac failure may not be a significant factor in the rapid increase in blood concentration.

3) In the liver, flecainide is primarily metabolized by CYP2D6. Clinically important variants of CYP2D6 are CYP2D6*2, *3, *4, *5, *10, *17, and *41. Among these, *10 is the most prevalent among Japanese individuals [4]. CYP2D6*10, which is mainly responsible for intermediate metabolism, appears to play an important role in the treatment of Japanese patients [5]. However, we did not assess these genetic polymorphisms in the patient, leaving this factor unknown. Given that flecainide exhibits a 42% longer elimination clearance and a five-fold longer elimination half-life in patients with cirrhosis compared with that in healthy volunteers, being vigilant for increased blood levels of flecainide in individuals with severe cirrhosis is imperative [6]. In essence, when administering TDM to patients with cirrhosis, considering the prolonged elimination half-life is crucial, which extends the time required to reach a steady state. However, considering antiarrhythmic drugs such as flecainide, of which the main metabolic pathway involves CYP2D6, hepatic metabolism begins to decline notably in severe cirrhosis (Child-Pugh classification class C) [7]. Her Child-Pugh liver severity score was eight points, classified as moderate prognosis (class B), suggesting that hepatic metabolism may be less affected than in severe cirrhosis.

We conclude that the primary factor contributing to the prolonged half-life of flecainide in this case was the significant decrease in renal function. In the present case, neither the volume of distribution nor the area under the curve could be determined, given the inability to measure drug concentration in the blood during the early stages of administration (<24 h after dosing). However, several blood drug concentrations were measured so the half-life could be calculated. It is noteworthy that our results showed a considerable deviation from the pharmacokinetics outlined in the package insert. However, several blood drug concentrations were measured so the half-life could be calculated. Of note, our results showed a considerable deviation from the pharmacokinetics outlined in the package insert. However, given the nature of this study as a case report, further investigations with larger sample sizes are warranted to validate the relationship between the degree of renal dysfunction obtained and the changes in drug pharmacokinetics. Confirming detailed pharmacokinetics during the elimination phase, as in this case, and accumulating data on individual patients is crucial. Additionally, assessing blood levels in the early stages of drug administration (<24 h after dosing) and exploring the possibility of estimating total clearance from the elimination half-life in patients with various diseases would be valuable avenues for future research. Unfortunately, during the previous year, we did not measure flecainide blood levels. Therefore, we were not aware of the exact time when the blood levels were raised above the therapeutic range. Measuring the blood levels of drugs routinely is important. In addition, we were unable to test for genetic polymorphisms in CYP2D6 at our institution, which is a factor that cannot be neglected when considering the hepatic clearance of flecainide. We recommend that future studies focus on investigating CYP2D6 polymorphisms.

## Conclusions

Older adults, particularly those with diminished renal function, may experience substantial increases in drug blood concentrations compared with healthy adults. Emphasizing the pharmacokinetics of drugs during their elimination phase is crucial.
